# Pandemic Influenza Vaccines – The Challenges

**DOI:** 10.3390/v1031089

**Published:** 2009-12-03

**Authors:** Lars R. Haaheim, Abdullah S. Madhun, Rebecca Cox

**Affiliations:** 1 Myrdalskogen 95, N-5117 Ulset, Norway; 2 Influenza Centre, The Gade Institute, University of Bergen, Laboratory Building, 5th Floor, Haukeland University Hospital, N-5021 Bergen, Norway; E-Mails: abdullah.madhun@gades.uib.no (A.S.M.); Rebecca.Cox@gades.uib.no (R.C.)

**Keywords:** influenza, H5N1, H1N1, pandemic, vaccine, haemagglutinin (HA), neuraminidase (NA)

## Abstract

Recent years’ enzootic spread of highly pathogenic H5N1 virus among poultry and the many lethal zoonoses in its wake has stimulated basic and applied pandemic vaccine research. The quest for an efficacious, affordable and timely accessible pandemic vaccine has been high on the agenda. When a variant H1N1 strain of swine origin emerged as a pandemic virus, it surprised many, as this subtype is well-known to man as a seasonal virus. This review will cover some difficult vaccine questions, such as the immunological challenges, the new production platforms, and the limited supply and global equity issues.

## 1. Introduction

While most people had anticipated the next pandemic virus to have a novel HA subtype of avian origin (such as H5), the sudden emergence in 2009 and the rapid global spread of swine-origin influenza A (H1N1) virus (H1N1 pandemic virus) surprised many. The World Health Organization declared the pandemic (Phase 6) on June 11 after nearly 30,000 confirmed cases have been reported in 74 countries [1].

Preceding and following the transition to Phase 6, there was a flurry of publications on the genetic and antigenic characterization of the new virus [2–6]. There was a general consensus about the origin of the H1N1 pandemic virus, being the result of multiple reassortments steps between human H3N2, North American avian virus, and H1 from swine virus from the Eurasian lineage. The gestation period for this new H1N1 virus apparently started in the late 1990’s and culminated in swine at some undefined recent time-point prior to the emergence of the final virus in man. Also, the antigenic properties clearly indicated that the HA of the H1N1 pandemic strain was only distantly related to current seasonal H1N1 viruses [6]. This virus transmits easily and generally causes mild disease, although severe illness (respiratory distress) and apparent high fatalities were reported, mostly so during the first weeks in Mexico [7]. The worldwide spread of the pandemic influenza A (H1N1) and the continuous sporadic zoonotic cases of avian H5N1 increase the risk of the reassortment between the two viruses and therefore constitute an additional global threat.

Although influenza drugs remain a useful supplement, and occasionally also the only option for therapeutic and prophylactic intervention, emerging resistant strains could make them inefficient [8,9]. Whereas the H1N1 pandemic virus initially was shown to be resistant to the adamantanes, it was at first sensitive to the NA inhibitor drug oseltamivir, setting it aside from the seasonal H1N1 virus in recent years. A report by the WHO described sporadic cases of tamiflu resistant strains [10]. Whether such resistant strains will spread and become dominant remains to be seen, but it points to our current and very limited assortment of influenza drugs as a fragile part of our arsenal. Widespread indiscriminate and *ad lib* use of antivirals both in man and in animals in some countries will facilitate emergence of drug resistant strains. Also, and as a direct consequence of wasteful drug use, waterways could be contaminated and potentially set the scene for development of drug resistant influenza strains in aquatic birds [11]. This in itself poses a human health hazard, considering the potential exchange of influenza genes between the avian pool and human viruses. The large stockpiles of oseltamivir generated by many affluent countries as a major element of their pandemic preparedness, could potentially be of little use or even become ineffective.

Inactivated vaccines against influenza virus was first made during the 1940s, initially aimed at military personnel, but later also targeted for the civilian population, particularly the elderly and those at risk of serious illness and death. Our best option to mitigate the health and societal consequences of a pandemic is to have access to an efficacious vaccine in a timely manner [12]. Vaccination is the best option by which spread of a pandemic virus could be slowed down or halted and severity of disease reduced. Whereas the avian H5N1 virus, currently in WHO Pandemic Phase 3, belongs to a subtype against which virtually none has any pre-existing immunity, the H1N1 pandemic virus subtype has been circulating in man for the most parts of the 20^th^ century and until today. It has been shown that senior age cohorts of the population have some degree of immunity to the H1N1 pandemic virus [13]. This difference in pre-existing immunity and degree of previous priming will of course affect the vaccination strategies, as well as the dosage and number of immunizations needed to elicit the minimally required levels of vaccine-induced serum antibodies. Furthermore, neither for the H5N1 nor for the H1N1 pandemic virus do we know to what extent any vaccine-induced subtype priming will lessen the clinical impact when subsequently infected by the pandemic virus. Several new adjuvants have been clinically tried and licensed in Europe during the recent years [14,15]. Especially for H5N1 vaccines, oil-in-water based emulsions show great promises, whereas split or subunit formulations in combination with alum mainly fall short. Whole virion formulations, and also virus-like-particles (VLPs), perform well.

To supply enough tailor-made pandemic vaccines to the entire world’s population is a formidable challenge to global equity. Both the limited supply and the cost of vaccines will set the poor and non-industrialized countries in a difficult position. The lack of adequate health care infrastructure in the third world is an additional concern

## 2. Influenza Vaccines

### 2.1. Background; Seasonal Vaccines

Influenza vaccination is the most effective prophylactic measure to prevent morbidity and mortality (reviewed in [16]). There are two main types of influenza vaccine available; inactivated vaccine delivered deep subcutaneously or intramuscularly and live attenuated vaccine administered intranasally. Parenterally administered inactivated influenza vaccines have been used for many decades and extensive information is available on their quality and safety, being about 60–80% effective in preventing disease against homologous or closely related strains. In some cases, vaccination may not prevent influenza morbidity, but rather reduce its severity and duration. Inactivated vaccines are available in whole, split (chemically disrupted), subunit (purified surface glycoproteins) and virosomal/virus-like particles (VLPs) containing surface glycoproteins formulations.

Current inactivated vaccines are mostly produced by propagation in embryonated hens’ eggs. The allantoic fluid is harvested, and the virus is concentrated and highly purified, then inactivated by formaldehyde or beta-propiolactone, or eventually by the detergent disruption procedure itself. The availability of embryonated hens’ eggs is a limiting factor in vaccine production and the global manufacturing capacity is not expected to meet pandemic vaccine demands. It is therefore important to develop dose-sparing strategies by using effective formulations and/or adjuvants. Some manufacturers are using cell cultures as vaccine substrate (MDCK, Vero, PerC6®), either as their only platform or as a supplement, allowing more expediently to scale up of production (Table 1).

The use of reverse genetics technology can save a considerable amount of time in generation of pandemic seed virus. Traditionally, influenza vaccine seed viruses have been made by classical reassortment by choosing a virus isolate which closely matches circulating strains, and introducing its surface glycoprotein gene segments (HA and NA) into the genetic background of the laboratory-adapted and high-yielding “PR8” virus, A/Puerto Rico/8/34 (H1N1). Seed viruses are produced by co-infection of the two “parent” viruses, and screening for the progeny of interest. This is a time-consuming process and not always successful. And even so, the growth characteristics may not be acceptable. The use of reverse genetics allows viruses to be constructed with a predefined set of genes, following combination of plasmid DNAs encoding separate RNA segments. Site-directed mutagenesis allows engineering of the DNA transcripts to create viruses altered in specific genes. Highly pathogenic avian strains can be attenuated to create a depathogenised virus, which can be used as a vaccine strain, e.g. NIBRG-14 derived from A/Vietnam/1194/2004 (H5N1), which has been extensively used in human clinical trials. However, some of the recent vaccine reassortants vaccine strains for the pandemic H1N1 virus, whether prepared by classical reassortment or by reverse genetics, have been reported to give low yields.

Live attenuated seasonal vaccines have been used in Russia since the 1970s and have been licensed for use in the USA since 2003. There are two strains A/Leningrad/134/17/57 (H2N2) and A/Ann Arbor/6/60 (H2N2), which have been used as donor strains. They are attenuated, genetically stable, non-transmissible, safe, immunogenic, and provide protective immunity. Importantly, live attenuated vaccines can be rapidly manufactured and provide more vaccine doses per egg than inactivated vaccines, and they are conveniently administered by intranasal spray devices. Live attenuated vaccines induce a good mucosal response, which upon challenge with field strains may limit their initial replication in the upper respiratory tract, the viral portal of entry. These vaccines perform well in children, reduce laboratory confirmed infection in adults, but are less immunogenic in the elderly. A comprehensive review of the current status of attenuated live influenza vaccines is given by Ambrose *et al.* [17].

### 2.2. Pandemic Vaccines

#### 2.2.1. Non-H1 pandemic vaccines

One of the most important interventions in pandemic control is to provide a safe and effective vaccine in a timely manner. Most of the extensive development work on pandemic vaccines has focused on the H5 subtype, although some trials have used other avian subtypes such as H7 and H9, as well as the historic human subtype H2N2 (Table 1).

As reported by the WHO there were in 2008 more than 70 registered clinical trials of candidate pandemic influenza vaccines, less than 10 have used alternative production platform such as DNA vaccines, recombinant proteins made in *E. coli* and by engineered baculovirus in insect cells [18]. The vaccines have been reported as safe and well tolerated. The majority of the trials have been performed in young healthy adults, although recent trials have involved the elderly and young children. There are a number of vaccine manufactures having been granted regulatory approval for their H5N1 vaccines in Europe, USA and Australasia. The experience gained in development of pandemic vaccines to date has provided essential information on formulation, dosage, and use of adjuvants, and has saved valuable time in preparing a vaccine against the pandemic (H1N1) 2009 virus. The hope is that this will have an important impact on the public health particularly if a safe, immunogenic and appropriately formulated vaccine can be rapidly prepared in sufficient quantities. The use of a single dose regimen with a novel avian subtype is not sufficient for an immunologically naïve population, while a two dose vaccination course more often results in sero-conversion and fulfilment of the European CHMP (The Committee for Medicinal Products for Human Use) requirements [19–21].

A two-dose regimen of Vero cell grown whole H5N1 virus vaccine (7.5 or 15 μg HA) elicited good serum antibody responses in adults against antigenically diverse H5N1 virus strains. However, whilst whole virus vaccines have proved more immunogenic in unprimed adults than split or subunit vaccines, the majority of manufacturers are technically unable to convert their current split or subunit vaccine production process to whole virus formulations [21,22]. Current seasonal influenza vaccines contain 15 μg HA from each of the three seasonal strains, with a total of 45 μg HA. In contrast, very high antigen doses (up to 90 μg HA) were required for non-adjuvanted split virus or recombinant pandemic H5 vaccines to elicit an antibody response sufficient to meet the licensing criteria [23,24]. This underlines the need for effective adjuvants to enhance the immune response to split or subunit pandemic influenza vaccines. Some of the best candidate vaccine formulations currently available are adjuvanted or whole virion vaccines (reviewed in [25–27]).

Aluminium salts have so far been the most commonly used adjuvant, as they are non-proprietary and inexpensive, having been used in childhood vaccination programs for many years with an excellent safety record. For pandemic vaccines against avian HA, aluminium salts have only been shown to modestly augment the antibody response in adults after two doses of candidate pandemic vaccines; H5N1 influenza split virus vaccine (30–45 μg HA) [28, 29], H7N1 split virus vaccine (12–24 μg HA) [30], or low dose of H9N2 whole virus vaccine (1.9 μg HA) [19,22]. In children from 6 months to 9 years of age two doses of aluminium adjuvanted H5N1 split virus vaccine (30–45 μg HA) were strongly immunogenic [31]. However, other trials found that aluminium adjuvant did not significantly enhance the antibody response to candidate H5 vaccines [32–34].

Some of the most promising adjuvants are the proprietary oil-in-water emulsion based systems such as MF59 (Novartis) and ASO3 (GSK), which have been found to greatly enhance both homologous and cross reactive antibody responses after H5 vaccination even at lower antigen concentrations (3.8–7.5 μg HA) [21,32,35–38]. In a direct side-by-side comparison of H5N1 vaccine adjuvanted with aluminium salts and MF59, the latter was found to outperform aluminium [32]. In prime boost studies with 2–3 doses of vaccine based on H5N3 clade 0 and a booster dose up to 8 years later with one dose of MF59 adjuvanted vaccine with clade 1 H5N1 vaccine induced a rapid increase in antibody response one week after vaccination which was broadly cross reactive against H5N1 variants [39,40]. The AS03 adjuvant allowed dose sparing down to 3.8 μg HA for the A/Vietnam/1194/04 H5N1 vaccine. This vaccine has undergone extensive clinical trials including in children and the elderly, and is now licensed in Europe. Formulation with AS03 adjuvant induced an effective homologous and cross clade immune response [37,41]. A more recently developed oil-in-water adjuvant is the AFO3 (sanofi), resulting in virtually similar dose-sparing and cross-reactivity, with more than 80% seropositivity after 2 doses of 3,75 μg and high degree of cross-clade cross-reactivity [42]. Also, preliminary results from an ongoing phase I clinical trial of virosomal H5N1 vaccine with a 3^rd^ generation ISCOM™ adjuvant have shown equal or greater ability to dose-sparing than oil-in-water emulsion systems, as well as high frequencies of polyfunctional Th1 CD4 cells. Data from the corresponding pre-clinical trial has been published [43].

Live attenuated influenza vaccines (LAIV) seed strains have been produced for a number of potential subtypes (H2, H4-16), as summarized in [44]. The live attenuated H9N2 vaccine was found to be immunogenic in seronegative adults. However, clinical trials of other avian live attenuated vaccines using the attenuated A/Ann Arbor/6/60 (H2N2) as partner, have found that the vaccine strains are further attenuated and have lower infectivity and consequently less immunogenic than seasonal H1N1 and H3N2 strains [45]. In contrast, two doses of an H5N2 vaccine produced with the A/Leningrad/134/17/57 (H2N2) as the donor strain, elicited good serum and local immune responses [46]. In further trials, two vaccine doses have been administered at a 10 days interval instead of 21 days, and this increased the immunogenicity of the vaccine to homologous and heterologous strains (as reported in [44]). Although a recipient of an attenuated live vaccine virus is usually not contagious for others, there is always a theoretical risk of reassortment between other influenza viruses from man and/or animal sources. This elusive potential hazard should be weighed against the mostly excellent immune response, particularly mucosal and cytotoxic immunity, elicited by live vaccines and the potential to manufacture large quantities of vaccine within a short time span.

#### 2.2.2. H1N1 pandemic vaccines

For the current H1N1 situation, the developments had followed a quick pace, a stark contrast to the drawn-out avian H5N1 threat that has been with us for more than a decade. The H1N1 pandemic virus was detected in Mexico in March 2009 and spread throughout Mexico and the United States in April, and subsequently beyond these two countries. During the months of May and June early online publications on the origin and the pandemic potential of the novel H1N1 became available [3–6]. WHO declared the pandemic (Phase 6) on 11 June 2009.

On 22 May 2009, using pre- and post-vaccination sera from individuals having received previous seasons’ influenza vaccines, it was possible to gauge the cross-immunity to the H1N1 pandemic strain in various age groups by haemagglutination-inhibition and microneutralization tests [13]. It was demonstrated that, although about a third of elderly vaccinees (60+) had pre-vaccination antibodies against the pandemic H1N1 strain, the frequency of seropositives only rarely and marginally increased after vaccination with recent years’ seasonal vaccines. For younger age groups, and particularly for children, antibodies to the novel H1N1 virus was in essence absent, and no apparent recall of cross-reactive antibodies was detected. It was concluded, although large segments of the populations have experienced the H1N1 subtype through previous infections or vaccinations,- that a vaccine against the pandemic H1N1 strain was needed. This study has been extended, including adjuvanted seasonal vaccines, and showed no additional cross-reaction to the pandemic H1N1 virus. Interestingly, using serum archives from the vaccine campaign in 1976 against the A/New Jersey (H1N1) swine influenza, a significant proportion (54%) sero-converted against the H1N1 pandemic virus [47].

Still, it has been speculated whether seasonal vaccine will give some degree of protection against the pandemic H1N1 virus. As this virus belongs to a subtype that has circulated throughout decades, it is an intriguing question whether seasonal vaccines, containing H1N1 virus, will confer any immunity to the pandemic virus. In a matched case-control study from Mexico it was stated that some protection, particularly against severe outcome, was associated with being vaccinated against seasonal influenza [48], whereas a report from the CDC argues that such an effect has not been convincingly proven [49].

Data from only a limited number of clinical trials have now been published [50–52]. The frequency of pre-existing antibodies varied somewhat in these trials. In an Australian study of subjects aged 18–64 years about 30% (irrespective of age) had pre-vaccination haemagglutination-inhibition titres ≥ 1:40, whereas a Chinese trial only showed low frequencies (1–6%) throughout all age groups [50,51]. In a European trial of subjects aged 18–50 years about 16% had protective pre-vaccination antibody levels [52]. Considering the poor reproducibility and variable sensitivity of current serological methods (haemagglutination-inhibition and microneutralization tests), and data from the three mentioned clinical trials, as well as the studies based on sero-archives [13,47], the degree of pre-pandemic herd immunity is not clear and whether there are major age and geographical differences. A more fundamental approach to estimate any potential pre-existing immunity has been published, using an influenza epitope sequences database and scanning the variability of T- and B-cell influenza H1N1 epitopes [53]. It was found that only 31% (8/36) of B-epitopes of the pandemic virus were conserved in relation to recent seasonal H1N1 strains, with only 17% conservation in the HA molecule. In contrast, and also demonstrated experimentally using peripheral blood lymphocytes from adults, approximately 70% of CD8^+^ cells recognized invariant H1N1 epitopes. While T-cell immunity will not block viral infection, it will assist in dampening the clinical outcome. Considering the different age-related pathology of the current pandemic virus, it would have been useful also to investigate lymphocyte from children and the elderly in the same manner.

In the Australian trial, using one dose of non-adjuvanted split egg-grown vaccine containing 15 μg HA, close to 97% of subjects attained protective antibody levels after 3 weeks [50]. No differences between age groups were observed, nor did the response improve by using 30 μg HA per dose. These are very encouraging results, clearly indicating that a dose reduction below 15 μg HA may be considered. Also, and equally encouraging for this non-adjuvanted vaccine, the scheduled 2^nd^ dose may not be immediately required.

In the Chinese study it was used egg-grown alum-adjuvanted and non-adjuvanted split vaccine formulations containing varying doses of HA, ranging from 7.5μg to 30μg HA [51]. As has been sporadically observed previously, the use of alum in this trial did not improve the vaccine performance [32–34]. For the 15μg non-adjuvanted treatment group about 87% of all subjects elicited haemagglutination-inhibition antibody titres ≥ 40 at three weeks post-vaccination, increasing to 97% at five weeks after the second dose. The age group 12–60 years gave the most vigorous response, with over 97% of subjects reaching protective levels of haemagglutination-inhibition antibodies after one dose. The authors conclude that one dose of 15μg may be sufficient to provide a protective immune response in the age group 12–60 years, whereas they argue that younger children and the elderly may require two doses. The 7.5μg treatment group of the study did not have a non-adjuvanted arm. In a dose-sparing perspective and considering the data presented, it is tempting to speculate whether a 7.5 μg formulation without alum might have elicited a satisfactory response.

The European study [52] tested subjects in the age group 18–50 years given MDCK cell-grown virus formulated as a subunit vaccine adjuvanted with MF59. The trial had a complex design and only the adjuvanted 7.5 μg and 15 μg (=2 × 7.5 μg) data were presented. Both vaccine strengths induced protective levels of anti-haemagglutination-inhibition antibodies (80% and 92%, respectively) and neutralizing antibodies (100% and 100%, respectively) at day 21 post-vaccination. Although the results from all the treatment groups were not presented in this interim report, and not knowing the results from the non-adjuvanted formulations, one may speculate whether a 7.5 μg adjuvanted dose may be adequate. It should be noted that the GSK pandemic H1N1 vaccine, adjuvanted with ASO3, and now in use in many countries, contains 3.8 μg HA per dose.

### 2.3. Immunological Challenges

In contrast to the current H1N1 pandemic, an eventual pandemic caused by the avian H5N1 would have met a virtually immunologically naïve global population and potentially caused a very high number of excess deaths. The compiled data for the zoonotic H5N1 cases suggest an overall case-fatality rate of 59% (262/442), with Indonesia standing out with 82% (115/141) [54]. In the event of a sustained human-to-human spread, it must be assumed that the fatality rates will not reach such high levels. The antigenic and genetic diversity of the avian H5N1 is a particular challenge for vaccine development. There are substantial antigenic differences between clades and subclades having consequences for vaccine development. So far, the WHO advocates that viruses from clades 1 and 2 should be used as vaccine seeds [55]. Whether or not an H5N1 pandemic will occur we cannot know, of course, nor is it possible to predict which clade and strain will eventually emerge as the pandemic virus. This makes pre-pandemic preparations of trial lots of H5N1 vaccines complicated. On the other hand, this uncertainty has stimulated research and development work, particularly when it comes to generating a strong cross-reactive vaccine response. Consequently, it has highlighted the importance of generating cellular immunity, predominately elicited against the internal and less variable antigens of the virus, namely the type-specific NP and M1 proteins, thus eliciting an immune response being more robust to antigenic changes.

The H1N1 pandemic situation is in many ways the complete opposite to the H5N1 threat. While the number of highly lethal zoonotic H5N1 cases has slowly continued to increase throughout the recent years, the virus still has not adapted sufficiently to allow effective human-to-human transmission to sustain community-level outbreaks. For the H5N1 virus we have been in the WHO pandemic phase 3 for six years (since 2003). It may well be that pandemic viruses not necessarily must emerge via an human adaption process from a zoonotic case, or as a result of a singular reassortment event between an animal and human strain. There could also be several reassortment incidents, all spaced in time and place, and involving different animal species, as highlighted for the recent H1N1 pandemic by Smith *et al*. [56]. We should therefore not lose sight of the H5N1 threat.

Ideally, a candidate pandemic influenza vaccine should elicit a rapid and strong humoral and cell-mediated immune response, and ideally also a strong mucosal response, which are long-lasting and exhibit broad cross-reactivity against drifted strains (within and across different clades). To date, most published results from animal studies and clinical trials of parenteral pandemic avian influenza H5N1 virus vaccines have highlighted the need for high antigen doses or an effective adjuvant to elicit a satisfactory antibody response, as highlighted by Hehme *et al.* [19] and Treanor *et al.* [23,24].

It is widely accepted that serum antibodies directed against the HA correlate with protection against seasonal influenza. However, there is convincing evidence, that both CD4^+^ T helper cells and CD8^+^ cytotoxic T lymphocytes (CTL) may play an important role in controlling viral infection and reduce the severity of disease and decrease mortality [57,58]. It has been well established that purified viral proteins formulated with oil-in-water adjuvants, stimulate the Th1 arm of the post-vaccination response. The same is also seen for inactivated whole virion vaccines and VLP formulations. It is assumed that viral proteins are entering the antigen-presenting cell via the endosomal pathway into the cytosol for subsequent presentation of viral peptides on MHC Class I, thus mimicking the infectious process. Also vaccine formulations with ISCOMs have been extensively studied and demonstrated CTL responses (reviewed by Rimmelzwaan *et al*. [59]. Therefore, the use of whole virion vaccines formulations, VLPs and/or the use of Th1 stimulating adjuvants would greatly improve vaccine performance.

Current vaccine seeds, whether prepared by classical reassortment or by reverse genetics, will, - by design,- not contain the NP and M1 proteins from the epidemic/pandemic strain, but from an historic laboratory reassortment partner. One may speculate whether this could constitute a disadvantage when aiming at stimulating a more authentic CTL response. One manufacturer (Baxter) uses the unmodified field virus as seed strain for their cell-grown inactivated whole virus vaccines, and in the context of cellular response this approach may well turn out to be especially advantageous.

The three pandemic H1N1 clinical studies just published [50–52] are mostly presenting preliminary results. The finer details of the immune response, e.g. Th1/Th2 profile and cross-reactions with emerging variant strains, will hopefully be presented in due time. Regarding the need for one or two doses, even if one dose satisfied licensing requirements, only follow-up studies of longevity of antibodies and humoral and eventual cellular memory will clarify this issue.

The poor immunogenicity of pandemic vaccines against H5 and H7 avian subtypes appears not to have repeated itself for the currently used H1N1 pandemic vaccines. This is a comforting finding.

Mucosal immunity may also provide protection against infection *per se* and not only protection against illness. Therefore, needle-free mucosal influenza vaccine is an attractive approach, which may provide immunity at the portal of virus entry. Additionally, mucosal IgA has broader specificity than serum IgG and may provide better cross-clade protection against drifted influenza strains [60,61]. However, although demonstrating stimulation of secretory IgA when using intranasal route for administration of influenza antigen alone, *i.e.* a non-live formulation, the immunogenicity is frequently poor [62–64]. Use of an appropriate nasal adjuvant could improve immunogenicity and thus reduce the required dosage [65]. One should, however, not forget the many cases of Bell’s palsy associated with a recent clinical trial involving intranasal vaccination [66].

Therefore, safe and effective mucosal adjuvants are urgently needed. A recent murine study with sublingual administration of an adjuvanted H1N1 vaccine has shown good mucosal immunity reflected by high IgA levels in the respiratory tract and provided protection against viral challenge [67]. Sublingual administration of a pandemic vaccine may significantly contribute to the pandemic preparedness through its ease of administration and better public compliance. Additionally, sublingual vaccines are less likely to induce neurological disorders and most probably a safer alternative than the intranasal vaccine for mucosal delivery, avoiding the potential exposure of the olfactory bulb [66]. Provided not excessive use of antigenic material is required, this alternative route should have much to offer and warrants further studies.

Few reports have addressed the long-lasting immunity and memory response after pandemic influenza vaccines [38,40]. Most of the published data evaluated the response in the immediate weeks after vaccination, which may underestimate the long-term protection against influenza viruses. However, it is difficult to assess the precise longevity of vaccine-induced immunity in the field, as influenza viruses undergo a continual antigenic variation (‘antigenic drft’). It is for this reason seasonal vaccination is recommended annually. Therefore, long-term humoral and cellular immunity after pandemic vaccines should be evaluated considering that more than one pandemic wave may occur [68].

### 2.4. New Production Platforms

Despite recent decades’ many new technical-scientific advances, the influenza vaccine production platforms have essentially remained unchanged [69]. While egg-grown influenza vaccines in the 1950’ties where initially rather reactogenic, subsequent introduction of zonal centrifugation purification steps, and also the introduction of detergent-split virions, greatly reduced its reactogenicity. Throughout the later parts of the 20^th^ century the global production capacity, and the actual use of vaccine against seasonal influenza, has steadily increased. Most manufacturers still grow viruses in embryonated hens’ eggs, whereas a small number of companies use cell cultures (Vero, MDCK, PER.C6®), either as their only substrate or as a supplement to eggs. The use of embryonated eggs is a critical point, as an emergency decision to scale up the production may not easily be accomplished as quality assured eggs must be ordered many months in advance. Adding to this complexity is the season-dependent quality of embryonated eggs, affecting the resultant viral yield. Production platforms based on approved cell lines are more controllable and more straightforward to scale up. One should not forget, however, that the availability of approved sites for bioreactors might potentially be a limiting factor. The fragile egg-based system is demonstrating its shortcomings during times of a pandemic urgency, such as today. Also, high pathogenic avian influenza viruses may add to the complexity by making the egg-laying flocks more vulnerable. Adding to this is the rare, but very critical consequences for vaccine output, when bacterially contaminated eggs are entering the production lines. This was clearly demonstrated in 2004 [70]. There is therefore a need for several new, or at least supplementary, vaccine production systems being more robust, more flexible and easier to scale up.

While some plasmid DNA vaccines have shown some promising results in animal models, there is still a way to go for generating effective human DNA vaccines. There is also a public acceptance issue to consider. In contrast, recent years industrial use of recombinant DNA technology, allowing generation of viral proteins to be made in large quantities in various host cells, hold promise for future vaccine technologies. In the context of influenza both DNA vaccines and recombinant protein preparations produced in insect and *E.coli* cell cultures have been used, some of them have got into clinical trials [71–75]. Although the strategy of producing edible vaccines has not been abandoned, the transient expression of engineered vaccine proteins in plant cells has shown promise. Such low cost systems are considered safe and can easily be scaled up (see review [76]). None of these new platforms have hitherto made any noteworthy contribution to the global annual influenza vaccine output. This will probably change in the years to come. Still, of the more than 70 pre-H1N1 registered trials with vaccines against pandemic influenza, several have used DNA vaccines, recombinant proteins made in *E. coli* and by engineered baculovirus in insect cells [18]. The most attractive property of the new production platforms is their claim to produce very large quantities of vaccine material within a short time. This is exemplified by a recent press release from VaxInnate Corp [77]. Using engineered bacterial flagellin, a TLR agonist, carrying viral sequences and targeting antigen presenting cells, they claimed that 300 millions H1N1 doses could be produced in weeks rather than months.

### 2.5. Limited Supply and Global Equity

For a pandemic, the ultimate objective is to produce enough vaccine to immunize the world’s entire population (6.7 billion people) with 2 doses, within 6–9 months, anticipating 5 μg HA/dose [78]. As mentioned previously, the total global output capacity for trivalent seasonal vaccines, requiring 15 μg HA for each strain, is estimated to reach approximately 1 billion doses by 2010 [79]. Assuming that all production capacity is completely switched to manufacture pandemic vaccines, and that 2 doses of 5 μg HA per dose is required, this will translate to approximately 4.5 billion immunization courses, representing about 67% of the global need. Such calculations will of course depend heavily on whether 2 doses are necessary for all age groups, which dose strength is required to satisfy licensing requirements, and whether dose-sparing adjuvants will be used. (Table 3). Considering the mixed type/subtype/strain influenza epidemiology during recent months, it is reasonable to assume that both A/H3N2 and B viruses, and possibly also the seasonal A/H1N1 virus, will continue to circulate. The calculations presented in Table 3 are therefore only representing a best case and probably also an unlikely scenario where manufacturers only have to consider one influenza vaccine strain.

Most importantly, for these calculations it is assumed that the growth characteristics of the seed virus will be about as good as recent years’ vaccine strains. WHO published an overview of viral characteristics with recommendations for vaccine development as early as 26 May, 2009 [80]. However, the growth characteristics of the pandemic H1N1 seed strains so far having being distributed to manufacturers have been questioned, thus the initial estimates of number of available doses may well be less than was optimistically suggested in Table 3. Still, there is substantial dose-stretching potential if the HA dosage can be reduced. Based on recent studies [50–52] it is also apparent that if certain age-cohorts only need one and not two vaccine doses, tentatively shown as 70% of the population in Table 2, there is a saving to be gained. Also, the use of intradermal immunization may offer savings in the order of about 80% in antigen usage [81–83]. When there is a global scarcity of vaccine, particularly when facing a pandemic, such an approach should be seriously considered. It should also be noted that the calculations in Table 3 show the theoretical capacity to provide a pandemic H1N1 vaccine for the global population, varying from 22% to full coverage. In reality, the situation will be quite different, since the doses will not be delivered in full at one specifically set date. According to calculations by the WHO approximately 50% of total output will be available within 6 months [84].

Based on currently used technologies there is a five-six month lead-time from having identified the pandemic strain to release of the first vaccine lots [85]. This includes generating seed virus (by classical reassortment or by reverse genetics), preparing reference sera and antigens for potency standardization purposes, optimizing manufacturing processes, bulk manufacture, quality control, vaccine filling, clinical studies and regulatory approval [86]. For the preparation of seed virus, the use of reverse genetics *in lieu* of classical reassortments, has the potential of cutting several weeks off this time-line. Furthermore, the licensing hurdle can be dramatically shortened if the manufacturer has submitted and received approval for a mock-up process with the same subtype (but a different strain) without any change of the manufacturing process.

Clearly, using classical production platforms the necessary total global influenza vaccine output could possibly be within reach within the next few years, but the long lead-time required to provide enough vaccine in time for the entire global population is not acceptable. Following the H1N1 pandemic some countries have activated their dormant contractual arrangements with the pharmaceutical industry for prioritized delivery of vaccines for their populations. Global solidarity issues will be raised, and rich countries with such exclusive contracts will be asked to share their vaccine allocations with less affluent countries. While national politicians no doubt will battle with this quandary, it is reassuring to know that several vaccine manufacturers have pledged donations of millions of doses of pandemic vaccine to the WHO for subsequent distribution to low-income countries. To what extent national authorities, particularly those hosting vaccine manufacturers, will activate emergency statutes and ban export of such a life-saving commodity before their domestic need is satisfied, remains to be seen. It will most certainly depend upon how the clinical pattern of the H1N1 pandemic develops, assuming that a less virulent strain will translate into more generosity

## 3. Conclusions

Currently, we are in a unique pandemic situation. While the avian H5N1 virus has been in WHO phase 3 since 2004, the newly emerged H1N1 virus spread rapidly and developed into a full pandemic (phase 6) within a couple of months. Whether this two-pronged threat is unique in a historical perspective is impossible to say, nor is it possible to know how this scenario will play out. On 11 June 2009, Dr. Margareth Chen, Director General of the World Health Organization, said during a press conference when the pandemic was declared: “*We are in the earliest days of the pandemic. The virus is spreading under a close and careful watch. No previous pandemic has been detected so early or watched so closely, in real-time, right at the very beginning.”* Modern biological sciences have made big strides allowing detailed and almost in real time generation of data to enable analyses of the genetic make-up of the novel strain and present evidence-based scientific opinions about the origin of the emerging virus.

During the pandemics of 1957 and 1968 the new pandemic virus replaced the preceding subtype, from H1N1 to H2N2 and from H2N2 to H3N2, respectively. The reappearance of H1N1 in 1977 did not replace the current H3N2, and the two influenza A subtypes have continued to co-circulate. It is not known how the current and mixed situation will turn out. Will the seasonal H1N1 virus disappear and be replaced by the pandemic H1N1 virus? Will the H3N2 virus continue to circulate? These are important questions, as it will have profound consequences for the formulations of seasonal influenza vaccines during the coming seasons.

Still, when it comes to chemo-prophylaxis and -therapy, science has not made giant leaps. Influenza drugs are available, and the adamantanes (amantadin and rimantadin) have been used against influenza A for more than four decades, whereas the last decade’s neuraminidase inhibitors are targeting the viral NA function both for A and B viruses. However, drug resistance could make them less useful or even redundant, as widespread indiscriminate and *ad lib* use of antivirals in some countries will facilitate emergence of drug resistant strains.

As for community mitigation, we have to rely on old and well-proven interventions, while recognizing that modern societies are extremely vulnerable to large-scale absenteeism from work, particularly in the health services, and that modern days industrial wheels require an uninterrupted global trade for supply of goods and raw materials. For the third world in particular, the health and societal consequences could be even more destructive, realizing that drugs and vaccines will be scarce or not available.

A tailor-made pandemic vaccine, *i.e.* using approved strains representing the actual pandemic virus, is our best prophylactic option. While for the avian H5N1 virus, it has been argued that the use of a prepandemic vaccine, say in the eventual phase 4 or 5, using a vaccine dose and formulation that elicits a high degree of cross-reaction between strains of different H5 clades, - is an attractive option. For the pandemic H1N1 virus, events developed so rapidly that only a vaccine based on a representative and widespread strain will be acceptable. We were not given the time to develop and license prepandemic vaccine. It is important that while tackling the current H1N1 pandemic, we should not lose sight of the H5N1 warning. It could adapt to humans and develop into a human pandemic strain, or it could reassort with current human strains. The H1N1 pandemic virus is in this respect a worrying partner.

There is much vaccine development work to be undertaken. We need new vaccines eliciting a better cellular response where safe adjuvants will be crucial, and we need vaccines generating a long-lived and strong mucosal response. We should increase our efforts to stretch a limited vaccine supply, and the emerging new production platforms will here have a pivotal rôle.

Unfortunately, all those in need of vaccine will not get it in time, as the primary limiting factors are manufacturing speed and volume. Some quantities of pandemic vaccine will be available before the winter season 2009/10 starts in the northern hemisphere, whereas the southern hemisphere has battled with the first pandemic wave without access to vaccine. While the antigenic and genetic make-up of the pandemic H1N1 virus has hitherto been remarkably stable, this could change. The spread of the H1N1 virus among overcrowded and destitute townships in metropolitan areas and in communities already burdened by HIV, malaria and tuberculosis, could facilitate change of viral pathogenicity and antigenicity. An increased case-fatality rate is therefore a real concern. It will also be a question whether the pandemic vaccines now being prepared will be antigenically well matched with new variant field strains that may emerge during the coming months. Still, the litmus test for global solidarity will be whether the rich countries of the world, as well as the pharmaceutical industry, in a timely fashion are willing to share or donate significant quantities of antivirals and pandemic vaccine doses to the poorer nations.

## Figures and Tables

**Table 1.** Main properties of the most widely used influenza vaccines.

Type of influenza vaccine	Substrate ^1)^	Route of administration	Immune response ^2)^	Immunological challenges	Comments
**Inactivated**				No apparent stimulation of mucosal immunity	
Whole virion	Embryonated eggs, cell culture	intramuscular	Strong, mixed Th1/Th2		Supply of embryonated eggs is a limiting factor. Cell-grown vaccine production more scalable.
Split			Mostly Th2	Should be adjuvanted	
Subunit (HA and NA)			Mostly Th2	Least immunogenic, should be adjuvanted	
**Live attenuated**	Embryonated eggs	intranasal	Humoral, cellular, mucosal	Should perform better in the elderly	Rapid and large vaccine output. Easily scalable production. Cannot be used in the very young and the immunocompromised.

1) MDCK (Madin Darby Canine Kidney cells), Vero (African green monkey kidney cells), PerC6^®^ (human embryonic retinal cells);

2) Th1 and Th2 are T-cell subsets heralding cellular and humoral response, respectively.

**Table 2.** Reassortant vaccine strains used in clinical trials of pandemic vaccines [18].

Virus strain	Subtype
A/Singapore/1/57	H2N2
A/duck/Singapore/97	H5N1
A/Viet Nam/1194/2004 (NIBRG-14), clade 1	H5N1
A/Indonesia/5/2005, clade 2.1	H5N1
A/duck/Potsdam/88/92 (H5N2) × A/Leningrad/134/17/57 (H2N2)	H5N2 (live)
A/chicken/Italy H7N1×PR8 (RD-3)	H7N1
A/chicken/British Columbia/CN-6/2004	H7N3
A/Hong Kong/1073/99	H9N2
A/chicken/HongKong/G9/97 (H9N2) × A/AnnArbor/6/60 (H2N2)	H9N2 (live)

**Table 3.** Different scenarios for global availability of (monovalent) pandemic vaccine 2010. Vaccine courses in millions.

	1 dose to all		2 doses to all		70% 1 dose, 30% 2 doses	
HA per dose	No of vaccination courses	Global coverage	No of vaccination courses	Global coverage	No of vaccination courses	Global coverage
15 μg	3,000	45%	1,500	22%	2,300	34%
10 μg	4,500	67%	2,250	34%	3,450	52%
5 μg	9,000	>100%	4,500	67%	6,900	>100 %

Calculations are based on an estimated global production capacity of trivalent seasonal influenza vaccines to be 1 billion doses in 2010, each with 15 μg HA, and a global population of 6.7 billion.

## References

[1] World Health Organization (WHO) World now at the start of 2009 influenza pandemic, 11 June 2009. http://www.who.int/mediacentre/news/statements/2009/h1n1_pandemic_phase6_20090611/en/index.html.

[2] Zimmer SM, Burke DS (2009). Historical Perspective - Emergence of Influenza A (H1N1) Viruses. N Engl J Med.

[3] Fraser C, Donnelly CA, Cauchemez S, Hanage WP, Van Kerkhove MD, Hollingsworth TD, Griffin J, Baggaley RF, Jenkins HE, Lyons EJ, Jombart T, Hinsley WR, Grassly NC, Balloux F, Ghani AC, Ferguson NM (2009). Pandemic Potential of a Strain of Influenza A (H1N1): Early Findings. Science.

[4] Smith GJ, Vijaykrishna D, Bahl J, Lycett SJ, Worobey M, Pybus OG, Ma SK, Cheung CL, Raghwani J, Bhatt S, Peiris JS, Guan Y, Rambaut A (2009). Origins and evolutionary genomics of the 2009 swine-origin H1N1 influenza A epidemic. Nature.

[5] Neumann G, Noda T, Kawaoka Y (2009). Emergence and pandemic potential of swine-origin H1N1 influenza virus. Nature.

[6] Garten RJ, Davis CT, Russell CA, Shu B, Lindstrom S, Balish A, Sessions WM, Xu X, Skepner E, Deyde V, Okomo-Adhiambo M, Gubareva L, Barnes J, Smith CB, Emery SL, Hillman MJ, Rivailler P, Smagala J, de Graaf M, Burke DF, Fouchier RA, Pappas C, Alpuche-Aranda CM, López-Gatell H, Olivera H, López I, Myers CA, Faix D, Blair PJ, Yu C, Keene KM, Dotson PD, Boxrud D, Sambol AR, Abid SH, St George K, Bannerman T, Moore AL, Stringer DJ, Blevins P, Demmler-Harrison GJ, Ginsberg M, Kriner P, Waterman S, Smole S, Guevara HF, Belongia EA, Clark PA, Beatrice ST, Donis R, Katz J, Finelli L, Bridges CB, Shaw M, Jernigan DB, Uyeki TM, Smith DJ, Klimov AI, Cox NJ (2009). Antigenic and Genetic Characteristics of Swine-Origin 2009 A(H1N1) Influenza Viruses Circulating in Humans. Science.

[7] Perez-Padilla R, De la Rosa-Zamboni D, Ponce de Leon S, Hernandez M, Quiñones-Falconi F, Bautista E, Ramirez-Venegas A, Rojas-Serrano J, Ormsby CE, Corrales A, Higuera A, Mondragon E, Cordova-Villalobos JA (2009). the INER Working Group on Influenza Pneumonia and Respiratory Failure from Swine-Origin Influenza A (H1N1) in Mexico. N Engl J Med.

[8] De Clercq E (2006). Antiviral agents active against influenza A viruses. Nat Rev Drug Discov.

[9] Oxford JS (2007). Antivirals for the treatment and prevention of epidemic and pandemic influenza. Influenza Other Respi Viruses.

[10] World Health Organization (WHO) Global Alert and Response, Pandemic (H1N1) 2009 briefing note 1, Viruses resistant to oseltamivir (Tamiflu) identified, 8 July 2009. http://www.who.int/csr/disease/swineflu/notes/h1n1_antiviral_resistance_20090708/en/index.html.

[11] Söderström H, Järhult JD, Olsen B, Lindberg RH, Tanaka H, Fick J (2009). Detection of the Antiviral Drug Oseltamivir in Aquatic Environments. PLoS One.

[12] Luke CJ, Subbarao K (2006). Vaccines for pandemic influenza. Emerg Infect Dis.

[13] Katz J, Hancock K, Veguilla V, Zhong W, Lu XH, Sun H, Butler E, Dong L, Liu F, Li ZN, DeVos J, Gargiullo P, Cox N (2009). Serum Cross-Reactive Antibody Response to a Novel Influenza A (H1N1) Virus After Vaccination with Seasonal Influenza Vaccine. MMWR Morb Mortal Wkly Rep.

[14] Guy B (2007). The perfect mix: recent progress in adjuvant research. Nat Rev Microbiol.

[15] Pashine A, Valiante NM, Ulmer JB (2005). Targeting the innate immune response with improved vaccine adjuvants. Nat Med.

[16] Cox RJ, Brokstad KA, Ogra P (2004). Influenza virus: immunity and vaccination strategies. Comparison of the immune response to inactivated and live, attenuated influenza vaccines. Scand J Immunol.

[17] Ambrose CS, Luke C, Coelingh K (2008). Current status of live attenuated influenza vaccine in the United States for seasonal and pandemic influenza. Influenza Other Respi Viruses.

[18] World Health Organization (WHO) Initiative for Vaccine Research (IVR), Tables on the Clinical trials of pandemic influenza prototype vaccines. http://www.who.int/vaccine_research/diseases/influenza/flu_trials_tables/en/index.html.

[19] Hehme N, Engelmann H, Künzel W, Neumeier E, Sänger R (2002). Pandemic preparedness: lessons learnt from H2N2 and H9N2 candidate vaccines. Med Microbiol Immunol.

[20] Stephenson I, Nicholson KG, Glück R, Mischler R, Newman RW, Palache AM, Verlander NQ, Warburton F, Wood JM, Zambon MC (2003). Safety and antigenicity of whole virus and subunit influenza A/Hong Kong/1073/99 (H9N2) vaccine in healthy adults: phase I randomised trial. Lancet.

[21] Stephenson I, Nicholson KG, Colegate A, Podda A, Wood J, Ypma E, Zambon M (2003). Boosting immunity to influenza H5N1 with MF59-adjuvanted H5N3 A/Duck/Singapore/97 vaccine in a primed human population. Vaccine.

[22] Hehme N, Engelmann H, Künzel W, Neumeier E, Sänger R (2004). Immunogenicity of a monovalent, aluminum-adjuvanted influenza whole virus vaccine for pandemic use. Virus Res.

[23] Treanor JJ, Wilkinson BE, Masseoud F, Hu-Primmer J, Battaglia R, O'Brien D, Wolff M, Rabinovich G, Blackwelder W, Katz JM (2001). Safety and immunogenicity of a recombinant hemagglutinin vaccine for H5 influenza in humans. Vaccine.

[24] Treanor JJ, Campbell JD, Zangwill KM, Rowe T, Wolff M (2006). Safety and immunogenicity of an inactivated subvirion influenza A (H5N1) vaccine. N Engl J Med.

[25] Stephenson I, Nicholson KG, Wood JM, Zambon MC, Katz JM (2004). Confronting the avian influenza threat: vaccine development for a potential pandemic. Lancet Infect Dis.

[26] Haaheim LR (2007). Vaccines for an influenza pandemic: scientific and political challenges. Influenza Other Respi Viruses.

[27] Keitel WA, Atmar RL (2007). Preparing for a possible pandemic: influenza A/H5N1 vaccine development. Curr Opin Pharmacol.

[28] Bresson JL, Perronne C, Launay O, Gerdil C, Saville M, Wood J, Höschler K, Zambon MC (2006). Safety and immunogenicity of an inactivated split-virion influenza A/Vietnam/1194/2004 (H5N1) vaccine: phase I randomised trial. Lancet.

[29] Nolan TM, Richmond PC, Skeljo MV, Pearce G, Hartel G, Formica NT, Höschler K, Bennet J, Ryan D, Papanaoum K, Basser RL, Zambon MC (2008). Phase I and II randomised trials of the safety and immunogenicity of a prototype adjuvanted inactivated split-virus influenza A (H5N1) vaccine in healthy adults. Vaccine.

[30] Cox RJ, Madhun AS, Hauge S, Sjursen H, Major D, Kuhne M, Höschler K, Saville M, Vogel FR, Barclay W, Donatelli I, Zambon M, Wood J, Haaheim LR (2009). A phase I clinical trial of a PER.C6 cell grown influenza H7 virus vaccine. Vaccine.

[31] Nolan T, Richmond PC, Formica NT, Höschler K, Skeljo MV, Stoney T, McVernon J, Hartel G, Sawlwin DC, Bennet J, Ryan D, Basser RL, Zambon MC (2008). Safety and immunogenicity of a prototype adjuvanted inactivated split-virus influenza A (H5N1) vaccine in infants and children. Vaccine.

[32] Bernstein DI, Edwards KM, Dekker CL, Belshe R, Talbot HK, Graham IL, Noah DL, He F, Hill H (2008). Effects of adjuvants on the safety and immunogenicity of an avian influenza H5N1 vaccine in adults. J Infect Dis.

[33] Ehrlich HJ, Müller M, Oh HM, Tambyah PA, Joukhadar C, Montomoli E, Fisher D, Berezuk G, Fritsch S, Löw-Baselli A, Vartian N, Bobrovsky R, Pavlova BG, Pöllabauer EM, Kistner O, Barrett PN, Baxter H5N1 Pandemic Influenza Vaccine Clinical Study Team (2008). A clinical trial of a whole-virus H5N1 vaccine derived from cell culture. N Engl J Med.

[34] Keitel WA, Campbell JD, Treanor JJ, Walter EB, Patel SM, He F, Noah DL, Hill H (2008). Safety and Immunogenicity of an Inactivated Influenza A/H5N1 Vaccine Given with or without Aluminum Hydroxide to Healthy Adults: Results of a Phase I–II Randomized Clinical Trial. J Infect Dis.

[35] Nicholson KG, Colegate AE, Podda A, Stephenson I, Wood J, Ypma E, Zambon MC (2001). Safety and antigenicity of non-adjuvanted and MF59-adjuvanted influenza A/Duck/Singapore/97 (H5N3) vaccine: a randomised trial of two potential vaccines against H5N1 influenza. Lancet.

[36] Stephenson I, Bugarini R, Nicholson KG, Podda A, Wood JM, Zambon MC, Katz JM (2005). Cross-reactivity to highly pathogenic avian influenza H5N1 viruses after vaccination with nonadjuvanted and MF59-adjuvanted influenza A/Duck/Singapore/97 (H5N3) vaccine: a potential priming strategy. J Infect Dis.

[37] Leroux-Roels I, Borkowski A, Vanwolleghem T, Dramé M, Clement F, Hons E, Devaster JM, Leroux-Roels G (2007). Antigen sparing and cross-reactive immunity with an adjuvanted rH5N1 prototype pandemic influenza vaccine: a randomised controlled trial. Lancet.

[38] Banzhoff A, Gasparini R, Laghi-Pasini F, Staniscia T, Durando P, Montomoli E, Capecchi PL, di Giovanni P, Sticchi L, Gentile C, Hilbert A, Brauer V, Tilman S, Podda A (2009). MF59-adjuvanted H5N1 vaccine induces immunologic memory and heterotypic antibody responses in non-elderly and elderly adults. PLoS One.

[39] Stephenson I, Nicholson KG, Hoschler K, Zambon MC, Hancock K, DeVos J, Katz JM, Praus M, Banzhoff A (2008). Antigenically distinct MF59-adjuvanted vaccine to boost immunity to H5N1. N Engl J Med.

[40] Galli G, Hancock K, Hoschler K, DeVos J, Praus M, Bardelli M, Malzone C, Castellino F, Gentile C, McNally T, Del Giudice G, Banzhoff A, Brauer V, Montomoli E, Zambon M, Katz J, Nicholson K, Stephenson I (2009). Fast rise of broadly cross-reactive antibodies after boosting long-lived human memory B cells primed by an MF59 adjuvanted prepandemic vaccine. Proc Natl Acad Sci U S A.

[41] Leroux-Roels I, Bernhard R, Gérard P, Dramé M, Hanon E, Leroux-Roels G (2008). Broad Clade 2 cross-reactive immunity induced by an adjuvanted clade 1 rH5N1 pandemic influenza vaccine. PLoS One.

[42] Levie K, Leroux-Roels I, Hoppenbrouwers K, Kervyn AD, Vandermeulen C, Forgus S, Leroux-Roels G, Pichon S, Kusters I (2008). An adjuvanted, low-dose, pandemic influenza A (H5N1) vaccine candidate is safe, immunogenic, and induces cross-reactive immune responses in healthy adults. J Infect Dis.

[43] Madhun AS, Haaheim LR, Nilsen MV, Cox RJ (2009). Intramuscular Matrix-M-adjuvanted virosomal H5N1 vaccine induces high frequencies of multifunctional Th1 CD4(+) cells and strong antibody responses in mice. Vaccine.

[44] Hayden FG, Howard WA, Palkonyay L, Kieny MP (2009). Report of the 5th meeting on the “Evaluation of pandemic influenza prototype vaccines in clinical trials.” World Health Organization, Geneva, Switzerland, 12–13 February 2009. Vaccine.

[45] Girard M, Palkonyay L, Kieny MP (2008). Report of the 4th meeting on the “Evaluation of pandemic influenza prototype vaccines in clinical trials.” World Health Organization, Geneva, Switzerland, 14–15 February 2008. Vaccine.

[46] Rudenko L, Desheva J, Korovkin S, Mironov A, Rekstin A, Grigorieva E, Donina S, Gambaryan A, Katlinsky A (2008). Safety and immunogenicity of live attenuated influenza reassortant H5 vaccine (phase I–II clinical trials). Influenza Other Respi Viruses.

[47] Hancock K, Veguilla V, Lu X, Zhong W, Butler EN, Sun H, Liu F, Dong L, DeVos JR, Gargiullo PM, Brammer TL, Cox NJ, Tumpey TM, Katz JM (2009). Cross-reactive antibody responses to the 2009 pandemic H1N1 influenza virus. N Engl J Med.

[48] Garcia-Garcia L, Valdespino-Gómez JL, Lazcano-Ponce E, Jimenez-Corona A, Higuera-Iglesias A, Cruz-Hervert P, Cano-Arellano B, Garcia-Anaya A, Ferreira-Guerrero E, Baez-Saldaña R, Ferreyra-Reyes L, Ponce-de-León-Rosales S, Alpuche-Aranda C, Rodriguez-López MH, Perez-Padilla R, Hernandez-Avila M (2009). Partial protection of seasonal trivalent inactivated vaccine against novel pandemic influenza A/H1N1 2009: case-control study in Mexico City. BMJ.

[49] Gargiullo P, Shay D, Katz J, Bramley A, Nowell M, Michalove J, Kamimoto L, Singleton JA, Lu PJ (2009). Effectiveness of 2008–09 trivalent influenza vaccine against 2009 pandemic influenza A (H1N1) - United States, May–June 2009. MMWR Morb Mortal Wkly Rep.

[50] Greenberg ME, Lai MH, Hartel GF, Wichems CH, Gittleson C, Bennet J, Dawson G, Hu W, Leggio C, Washington D, Basser RL (2009). Response after One Dose of a Monovalent Influenza A (H1N1) 2009 Vaccine -- Preliminary Report. N Engl J Med.

[51] Zhu FC, Wang H, Fang HH, Yang JG, Lin XJ, Liang XF, Zhang XF, Pan HX, Meng FY, Hu YM, Liu WD, Li CG, Li W, Zhang X, Hu JM, Peng WB, Yang BP, Xi P, Wang HQ, Zheng JS (2009). A Novel Influenza A (H1N1) Vaccine in Various Age Groups. N Engl J Med.

[52] Clark TW, Pareek M, Hoschler K, Dillon H, Nicholson KG, Groth N, Stephenson I (2009). Trial of Influenza A (H1N1) 2009 Monovalent MF59-Adjuvanted Vaccine -- Preliminary Report. N Engl J Med.

[53] Greenbaum JA, Kotturi MF, Kim Y, Oseroff C, Vaughan K, Salimi N, Vita R, Ponomarenko J, Scheuermann RH, Sette A, Peters B (2009). Pre-existing immunity against swine-origin H1N1 influenza viruses in the general human population. Proc Natl Acad Sci USA.

[54] World Health Organization (WHO) Global Alert and Response (GAR), Cumulative Number of Confirmed Human Cases of Avian Influenza A/(H5N1) Reported to WHO, 24 September 2009. http://www.who.int/csr/disease/avian_influenza/country/cases_table_2009_09_24/en/index.html.

[55] World Health Organization (WHO) Global Alert and Response (GAR), Antigenic and genetic characteristics of H5N1 viruses and candidate H5N1 vaccine viruses developed for potential use as human vaccines, February 2009. http://www.who.int/csr/disease/avian_influenza/guidelines/h5n1virus/en/index.html.

[56] Smith GJD, Bahla J, Vijaykrishnaa D, Zhanga J, Poona LLM, Chena H, Webster RG, Peiris M, Guana Y (2009). Dating the emergence of pandemic influenza viruses. Proc Natl Acad Sci USA.

[57] O’Neill E, Krauss SL (2000). Heterologous protection against lethal A/HongKong/156/97 (H5N1) influenza virus infection in C57BL/6 mice. J Gen Virol.

[58] Droebner K, Haasbach E, Fuchs C, Weinzierl AO, Stevanovic S, Büttner M, Planz O (2008). Antibodies and CD4(+) T-cells mediate cross-protection against H5N1 influenza virus infection in mice after vaccination with a low pathogenic H5N2 strain. Vaccine.

[59] Rimmelzwaan GF, Fouchier RA, Osterhaus AD (2007). Influenza virus-specific cytotoxic T lymphocytes: a correlate of protection and a basis for vaccine development. Curr Opin Biotechnol.

[60] Brandtzaeg P (2007). Induction of secretory immunity and memory at mucosal surfaces. Vaccine.

[61] Belshe RB, Mendelman PM, Treanor J, King J, Gruber WC, Piedra P, Bernstein DI, Hayden FG, Kotloff K, Zangwill K, Iacuzio D, Wolff M (1998). The efficacy of live attenuated, cold-adapted, trivalent, intranasal influenzavirus vaccine in children. N Engl J Med.

[62] Takada A, Matsushita S, Ninomiya A, Kawaoka Y, Kida H (2003). Intranasal immunization with formalin-inactivated virus vaccine induces a broad spectrum of heterosubtypic immunity against influenza A virus infection in mice. Vaccine.

[63] Waldman RH, Wood SH, Torres EJ, Small PA (1970). Influenza antibody response following aerosal administration of inactivated virus. Am J Epidemiol.

[64] Wright PF, Murphy BR, Kervina M, Lawrence EM, Phelan MA, Karzon DT (1983). Secretory immunological response after intranasal inactivated influenza A virus vaccinations: evidence for immunoglobulin A memory. Infect Immun.

[65] Tamura S, Ito Y, Asanuma H, Hirabayashi Y, Suzuki Y, Nagamine T, Aizawa C, Kurata T (1992). Cross-protection against influenza virus infection afforded by trivalent inactivated vaccines inoculated intranasally with cholera toxin B subunit. J Immunol.

[66] Mutsch M, Zhou W, Rhodes P, Bopp M, Chen RT, Linder T, Spyr C, Steffen R (2004). Use of the inactivated intranasal influenza vaccine and the risk of Bell’s palsy in Switzerland. N Engl J Med.

[67] Song JH, Nguyen HH, Cuburu N, Horimoto T, Ko SY, Park SH, Czerkinsky C, Kweon MN (2008). Sublingual vaccination with influenza virus protects mice against lethal viral infection. Proc Natl Acad Sci U S A.

[68] Taubenberger JK, Morens DM (2006). 1918 Influenza: the mother of all pandemics. Emerg Infect Dis.

[69] Ulmer JB, Valley U, Rappuoli R (2006). Vaccine manufacturing: challenges and solutions. Nat Biotechnol.

[70] Center for Infectious Disease Research & Policy (CIDRAP) (2004). Scrambling for vaccine: a sampling of responses. www.cidrap.umn.edu/cidrap/content/influenza/general/news/oct2804fluseries.html.

[71] Chen MW, Cheng TJ, Huang Y, Jan JT, Ma SH, Yu AL, Wong CH, Ho DD (2008). A consensus-hemagglutinin-based DNA vaccine that protects mice against divergent H5N1 influenza viruses. Proc Natl Acad Sci U S A.

[72] Goji NA, Nolan C, Hill H, Wolff M, Noah DL, Williams TB, Rowe T, Treanor JJ (2008). Immune responses of healthy subjects to a single dose of intramuscular inactivated influenza A/Vietnam/1203/2004 (H5N1) vaccine after priming with an antigenic variant. J Infect Dis.

[73] Treanor JJ, Schiff GM, Couch RB, Cate TR, Brady RC, Hay CM, Wolff M, She D, Cox MM (2006). Dose-related safety and immunogenicity of a trivalent baculovirus-expressed influenza-virus hemagglutinin vaccine in elderly adults. J Infect Dis.

[74] Huleatt JW, Nakaar V, Desai P, Huang Y, Hewitt D, Jacobs A, Tang J, McDonald W, Song L, Evans RK, Umlauf S, Tussey L, Powell TJ (2008). Potent immunogenicity and efficacy of a universal influenza vaccine candidate comprising a recombinant fusion protein linking influenza M2e to the TLR5 ligand flagellin. Vaccine.

[75] Song L, Nakaar V, Kavita U, Price A, Huleatt J, Tang J, Jacobs A, Liu G, Huang Y, Desai P, Maksymiuk G, Takahashi V, Umlauf S, Reiserova L, Bell R, Li H, Zhang Y, McDonald WF, Powell TJ, Tussey L (2008). Efficacious recombinant influenza vaccines produced by high yield bacterial expression: a solution to global pandemic and seasonal needs. PLoS One.

[76] Chichester JA, Haaheim LR, Yusibov V (2009). Using plant cells as influenza vaccine substrates. Expert Rev Vaccines.

[77] VaxInnate Corp (2009). VaxInnate Reports Positive Results from Preclinical Testing of Swine Flu Vaccine Developed Using Novel Technology. Press release.

[78] Kieny M-P WHO.

[79] World Health Organization (WHO) Projected supply of pandemic influenza vaccine sharply increases, 23 October 2007. http://www.who.int/mediacentre/news/releases/2007/pr60/en/index.html.

[80] (2009). Characteristics of the emergent influenza A (H1N1) viruses and recommendations for vaccine development. www.who.int/entity/csr/resources/publications/swineflu/H1N1Vaccinevirusrecommendation26May2009.pdf.

[81] Belshe RB, Newman FK, Cannon J, Duane C, Treanor J, Van Hoecke C, Howe BJ, Dubin G (2004). Serum Antibody Responses after Intradermal Vaccination against Influenza. N Engl J Med.

[82] Kenney RT, Frech SA, Muenz LR, Villar CP, Glenn GM (2004). Dose Sparing with Intradermal Injection of Influenza Vaccine. N Engl J Med.

[83] Auewarakul P, Kositanont U, Sornsathapornkul P, Tothong P, Kanyok R, Thongcharoen P (2007). Antibody responses after dose-sparing intradermal influenza vaccination. Vaccine.

[84] World Health Organization (WHO) Influenza A(H1N1) 19 May 2009 update. Meeting with Vaccine Manufacturers CEOs. http://www.who.int/vaccine_research/H1N1vaccines.pdf.

[85] World Health Organization (WHO) Initiative for Vaccine Research (IVR), 1 May 2009. http://www.who.int/vaccine_research/The5-6month.pdf.

[86] Wood JM, Robertson JS (2007). Reference viruses for seasonal and pandemic influenza vaccine preparation. Influenza Other Respi Viruses.

